# Herpetofauna Richness, Diversity, and Occurrence at the Northern Ecotone of Longleaf Pine

**DOI:** 10.1002/ece3.72041

**Published:** 2025-08-31

**Authors:** Julianne Jones, Dylan Bryant, Erik Yando

**Affiliations:** ^1^ Old Dominion University Department of Biological Sciences Norfolk Virginia USA; ^2^ The Longleaf Alliance Milton Florida USA; ^3^ Virginia Tech Department of Biological Sciences Blacksburg Virginia USA

**Keywords:** amphibians, landscape mosaic, latitudinal ecotone, longleaf pine, *Pinus palustris*, reptiles, species richness

## Abstract

Habitat suitability commonly differs between the core and periphery of ecosystems. The longleaf pine ecosystem is recognized for its value in providing habitat to a variety of specialist and endemic species, especially herpetofauna. However, at the northernmost extent of longleaf pine (in southeastern Virginia) little is known about the denizen reptiles and amphibians. Moreover, the remaining longleaf pine ecosystem at the latitudinal ecotone is fragmented amongst a mosaic of other ecosystems. We examined herpetofauna diversity, richness, and capture rates between four focal habitat types—mature longleaf pine, restored longleaf pine, maple‐gum swamp, and pocosin‐bog—in the Zuni Pine Barrens of southeastern Virginia. We present an examination of herpetofauna community in one of the only remnant stands of longleaf pine in Virginia. Our findings show that herpetofauna abundance and diversity were greatest in the maple‐gum swamp, but the mature longleaf and restored longleaf areas did maintain both generalist and fire‐tolerant species. Twenty‐five species were found in the focal habitats of our study, with another nineteen incidental species found in nearby adjacent areas, including both fire‐tolerant species and some species of special concern. This work highlights the variety of herpetofauna in this area and warrants further study to explore species diversity, abundance, ecotone impacts, and habitat preferences.

## Graphical abstract

The longleaf pine ecosystem is recognized for its value in providing habitat to a variety of specialist and endemic species, especially herpetofauna; however, at the northernmost ecotone of longleaf pine (in southeastern Virginia) little is known about the denizen reptiles and amphibians. We examined herpetofauna diversity, richness, and capture rates between four focal habitat types—mature longleaf pine, restored longleaf pine, maple‐gum swamp, and pocosin‐bog—in the Zuni Pine Barrens of southeastern Virginia. Our findings show that herpetofauna richness and diversity were greatest in the maple gum swamp, but the mature longleaf and restored longleaf areas maintained both generalist and fire‐tolerant species.

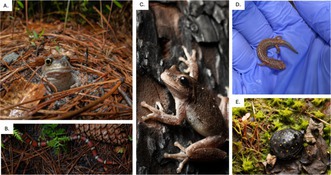

## 1 Introduction

The distributional extent of ecosystems may differ from the range limits of the specialist faunal and floral associates that inhabit them, with some communities and species only occurring in core areas due to climatic tolerances, dispersal limitations, competition, and habitat degradation (Guyer and Bailey 1993; Neilson 1993; Salisbury et al. 2012). At ecosystem margins, distinctive species assemblages may occur due to this mismatch of distributions, along with proximity to multiple different ecosystem patches (reviewed by Kark and Van Rensburg 2006). Distributional margins, hereafter ecotones, commonly result in gradual transitions, with dominant species decreasing in presence and their spatial extent increasing in patchiness compared to core areas (Gosz 1993). These ecotones provide opportunities for present species to interact with both novel species assemblages and adjacent ecosystem patches. Ecosystems that have a large geographic extent may see such a pattern, especially if core locations have high levels of specialists, multiple community types existing within an ecosystem, and the ecosystem's distributional ecotone maintaining a gradual and/or patchy boundary.

**FIGURE 1:**
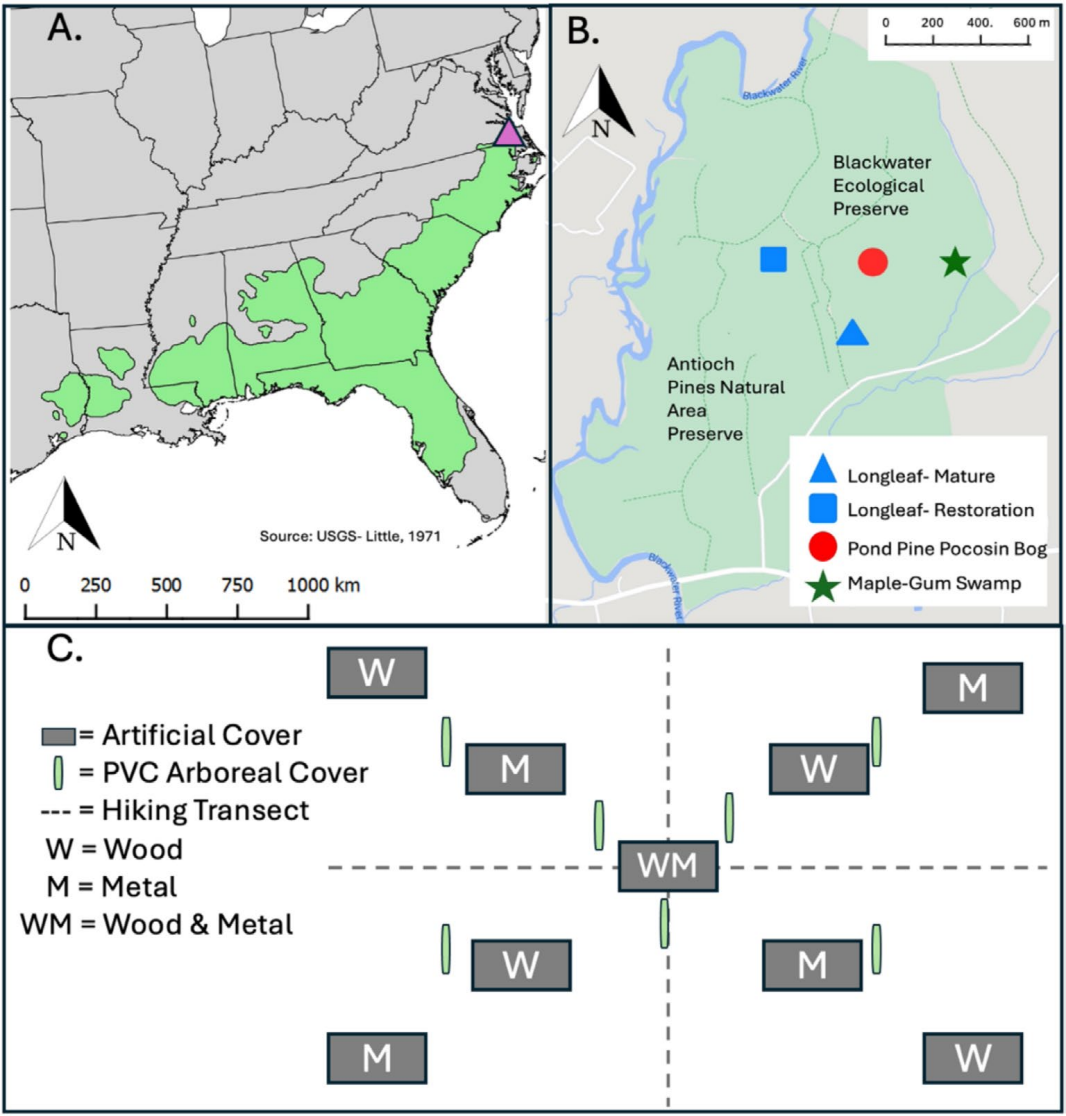
(A) Historical range of longleaf pine in the southeastern US (*Source:* USGS, Little 1971) with the study site shown in southeastern Virginia (purple‐triangle). (B) Location of longleaf mature (blue‐triangle), longleaf restoration (blue‐square), pond pine pocosin bog (red‐circle), and maple‐gum swamp (green‐start) at Blackwater Ecological Preserve and Antioch Pines Natural Area Preserve in Isle of Wight County, Virginia. (C) Plot set up of ground artificial cover (metal and/or wood), arboreal artificial cover (PVC), and hiking within each habitat type. Drawing is not to scale.

The longleaf pine ecosystem is a prime example of this phenomenon as they historically maintained a widespread distribution throughout the coastal plain of the southeastern US and exhibit multiple community types (e.g., flatwoods, montane, sandhills) (Frost et al. 1986; Peet 2006). Their original distribution ranged from east Texas to Florida in the south, extending throughout much of the coastal plain and as far north as southeast Virginia (Frost et al. 1986; Frost 1993) (Figure 1A). Longleaf pine ecosystems are fire‐adapted and home to a plethora of specialist flora and fauna, especially in core portions of their distribution in Mississippi, Alabama, Georgia, and Florida (Guyer and Bailey 1993; Walker 1995; Sorrie and Weakley 2006). Due to extensive human exploitation, land use change, and fire‐suppression, longleaf pine ecosystems have been greatly reduced in areal extent (Smith et al. 2000) and are now of great interest to restoration efforts throughout their historical range (McIntyre et al. 2018). In addition to the restoration of longleaf pine itself, interest in the restoration of specialist and associated species is becoming increasingly common (Camper 2005; Smith et al. 2017; Wenzel 2011; Weiss et al. 2019). For example, gopher tortoises ( *Gopherus polyphemus* ), reticulated flatwoods salamanders ( *Ambystoma bishopi* ), red‐cockaded woodpeckers ( *Leuconotopicus borealis* ), bobwhite quail ( *Colinus virginianus* ), and a range of rare plant species have been the subject of current conservation efforts (Camper 2005). At the longleaf pine northern latitudinal ecotone, beyond the range of many specialist associate species, there is also an interest in whole system restoration and conservation (Creighton et al. 2014), but there is a critical need to understand species presence, abundance, and how species differentially utilize longleaf pine ecosystems compared to adjacent habitats.

At the northernmost extent of longleaf pine, in southeastern Virginia, stands are made up of Longleaf Pine‐Savanna and Longleaf Pine‐Turkey Oak ecosystems (Musselman, Schafran, and Flanders 2018) that are markedly different in their community composition from their southern counterparts (Frost and Musselman 1987), namely due to their intermixing with loblolly and pond pine and their understory being dominated by ericaceous plants instead of grasses. This portion of the longleaf range is beyond the distribution of the commonly associated wire grasses (*Astrida* spp.) (Peet 1993). This expression of longleaf pine located primarily in the Blackwater River Basin is collectively known as the Zuni Pine Barrens and occurs in sandhill environments with significant efforts to reintroduce longleaf pine, fire regimes, and restore associated species (Creighton et al. 2014). While some studies have explored the unique floristic components of these northern longleaf forests (e.g., Frost and Musselman 1987; Lowenthal 1990; Bhuta et al. 2009; Johnsen et al. 2015), little work has occurred on the faunal components (Grefe 2010; Simons 2010), especially the herpetofauna communities. Beyond providing a needed baseline for this ecosystem margin, herpetofauna communities can serve as an important indicator for both ecosystem health and restoration (Litt et al. 2001; Waddle 2006) and are sensitive to multiple environmental gradients (Werner et al. 2007). Further, longleaf pine ecosystems support the greatest amphibian and reptile diversity of any temperate ecosystem in the core portions of their distribution, but Virginia's longleaf pine ecosystems are believed to be isolated from these specialists and overall lack thorough herpetological records (Rawinski and Ludwig 1992). A comprehensive assessment has the potential to inform short‐ and long‐term management, conservation, and restoration efforts while providing a needed starting point to study how present herpetofauna utilize these ecotonal environments.

In this study, we examined the presence and abundance of reptile and amphibian (hereafter herpetofauna) species within longleaf pine and adjacent ecosystems. Specifically, we examined (1) which herpetofauna are present in longleaf and adjacent areas at the longleaf pine latitudinal ecotone in southeastern Virginia? and (2) does habitat impact species richness and diversity? We hypothesized that generalist species would dominate the herpetofauna community present but expected a limited number of pinewoods and fire‐tolerant species to also occur due to ongoing prescribed fire management practiced in the area. Further, we hypothesized that, of all habitats examined, the adjacent maple‐gum swamp would have the greatest herpetofauna abundance and diversity due to its moist non‐acidic conditions, limited disturbance, and connectivity to other wetland ecosystems. This study importantly provides some of the first information on herpetofauna at the latitudinal longleaf pine ecotone in the southeastern US.

## 2 Methods and Materials

### 2.1 Sampling Design and Site Description

**FIGURE 2:**
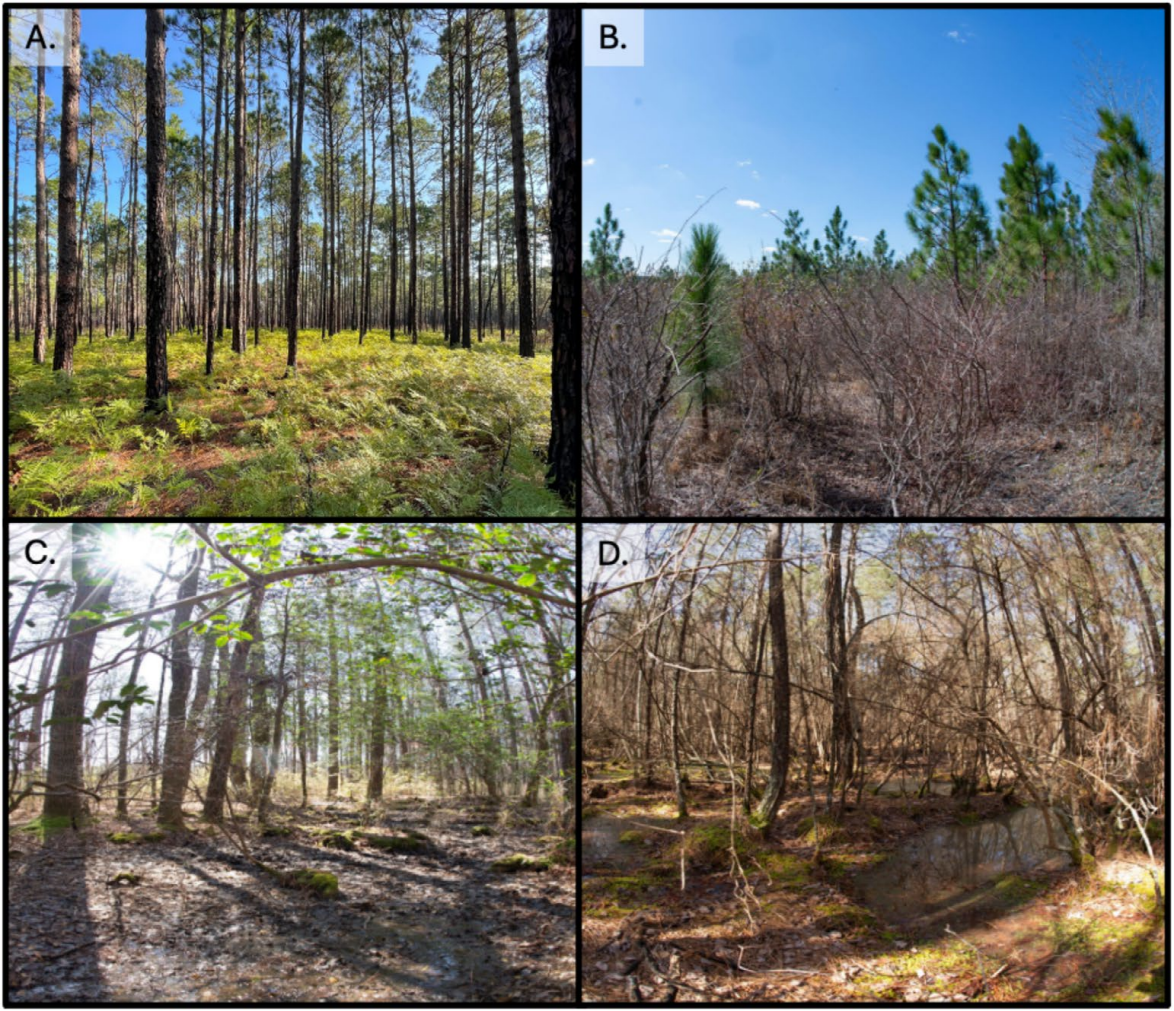
Representative images from all habitat types. (A) Longleaf‐Mature, (B) Longleaf‐Restoration, (C) Maple‐Gum Swamp, (D) Pocosin‐Bog. Images: J. Jones.

This work was completed within the Blackwater Ecological Preserve and adjacent Antioch Pines Natural Area Preserve located near Zuni, Virginia, a core portion of the Zuni Pine Barrens (Figure 1A,B). These sites have been regularly burned on a two to three‐year rotation since 1985, with varying severity based on soil moisture and fuel loads (Frost and Musselman 1987). Within this area we identified four distinct, adjacent habitat patches (mature longleaf savanna, an early successional longleaf restoration site, a pond pine pocosin‐bog, and a maple‐gum swamp) (Figures 1B and 2). Mature longleaf pine savanna was dominated by longleaf pine ( *Pinus palustris* ), with loblolly pine ( *Pinus taeda* ) and stunted turkey oak ( *Quercus laevis* ), alongside an understory of low ericaceous shrubs and ferns (Figure 2A). The longleaf restoration site was planted 13 years prior to this study (2010) and is now dominated by longleaf pine, turkey oak, and some sourwood ( *Oxydendrum arboreum* ), with an understory dominated by moderate height ericaceous shrubs, briars (*Smilax* spp.), and some graminoids (Figure 2B). The maple‐gum swamp is dominated by red maple, sweetgum (*Liquidambar styricaflua*), and black gum ( *Nyssa sylvatica* ) with some loblolly pine and oaks (*Quercus* spp.) on the margins, with an understory containing areas of dense switchcane (*Arundidaria tecta*) and Sphagnum mosses (Figure 2C). Finally, the pond pine pocosin‐bog is dominated by pond pine ( *Pinus serotina* ), stunted red maple ( *Acer rubrum* ), and contains multiple tall ericaceous shrubs (*Vaccinium* spp.), American Rattan ( *Berchemia scandens* ), and patches of Sphagnum mosses (Figure 2D).

We established a 15 × 15 m plot (225 m^2^) within each habitat type where we deployed five stacks of corrugated steel (0.61 × 1.22 m, two sheets per stack) and five pieces of plywood (0.61 × 1.22 m) to provide artificial cover for herpetofauna. Cover was placed diagonally within each plot in a 90° X‐shaped array across all habitat units in order to effectively survey the habitat type (Graeter et al. 2013) (Figure 1C). Cover objects were placed 5 m apart from each other and were alternated by type of material. (i.e., metal stack, plywood, metal stack, etc.). The center of each X‐array was made up of both plywood and metal, with plywood on the bottom layer (Figure 1C). In addition to artificial cover, seven tree‐mounted PVC pipes were deployed in each habitat unit for arboreal herpetofauna (reviewed by Glorioso and Waddle 2014). Four pipes were located on each of the inner corners of the X‐array, and the remaining three were placed randomly around the center of each habitat to fully sample core portions. Pipes were secured to the closest trees possible to inner‐corner cover objects, as well as the closest possible tree based on randomized values (Figure 1C). All PVC pipes were 5.08 cm (2″) in diameter and cut to length of 45.72 cm (18″). PVC pipes were capped on the bottom and small holes were drilled at 15 cm from the cap for drainage. Pipes were secured at 2 m from the ground. Additionally, two perpendicular transects (15 m) for the visual surveys were established through the middle of each plot (Figure 1C).

### 2.2 Herpetofauna Sampling

To estimate the presence and abundance of herpetofauna species, we conducted surveys of all artificial cover types, a subset of natural cover surveys, as well as hiking/visual encounter, night shining, and anuran vocalization surveys from February to July 2023 (Heyer 1994). Sampling was completed strategically throughout the season to limit sampling bias, with all areas being checked twice per week, with one daytime and one nighttime sampling effort for a total of 28 visits. For all surveys, the same number of hours was spent collecting data in each unit and in comparable conditions, with total person‐hours per unit and environmental conditions being recorded (Table S1). In addition to the sampling areas, we also collected information on individuals found by chance in areas outside of the established focal habitat units but still on the property to develop a comprehensive species list (Table S2).

Herpetofauna were identified to species or subspecies, if appropriate. We characterized species as generalists or pinewoods and fire‐tolerant species, with pinewoods and fire‐tolerant species being species that are closely associated with pine species and sandy soils, preferred by longleaf pine, for burrowing, foraging, and/or egg‐laying or those with a fire association (Guyer and Bailey 1993). Herpetofauna were not individually marked at capture due to the diversity of taxa and the duration of the sampling period. Capture counts between site visits are therefore inferred to be representative of the continuing suitability of a habitat for herpetofauna, rather than cumulative.

## 3 Data Analysis

As no true replicates existed in this study, analysis was limited to counts per unit effort and species richness, diversity, and coverage measures. Counts per unit effort were calculated by dividing the count data of each species by the total hours of effort and included all survey types within each habitat (visual, vocal, ground cover, arboreal cover). Species richness, diversity, and coverage measures were completed using species rarefaction and extrapolation curves and species coverage curves using the iNEXT package (Chao et al. 2014) in R and R Studio (v2023.12.1) (R Core Team 2024).

## 4 Results

**FIGURE 3:**
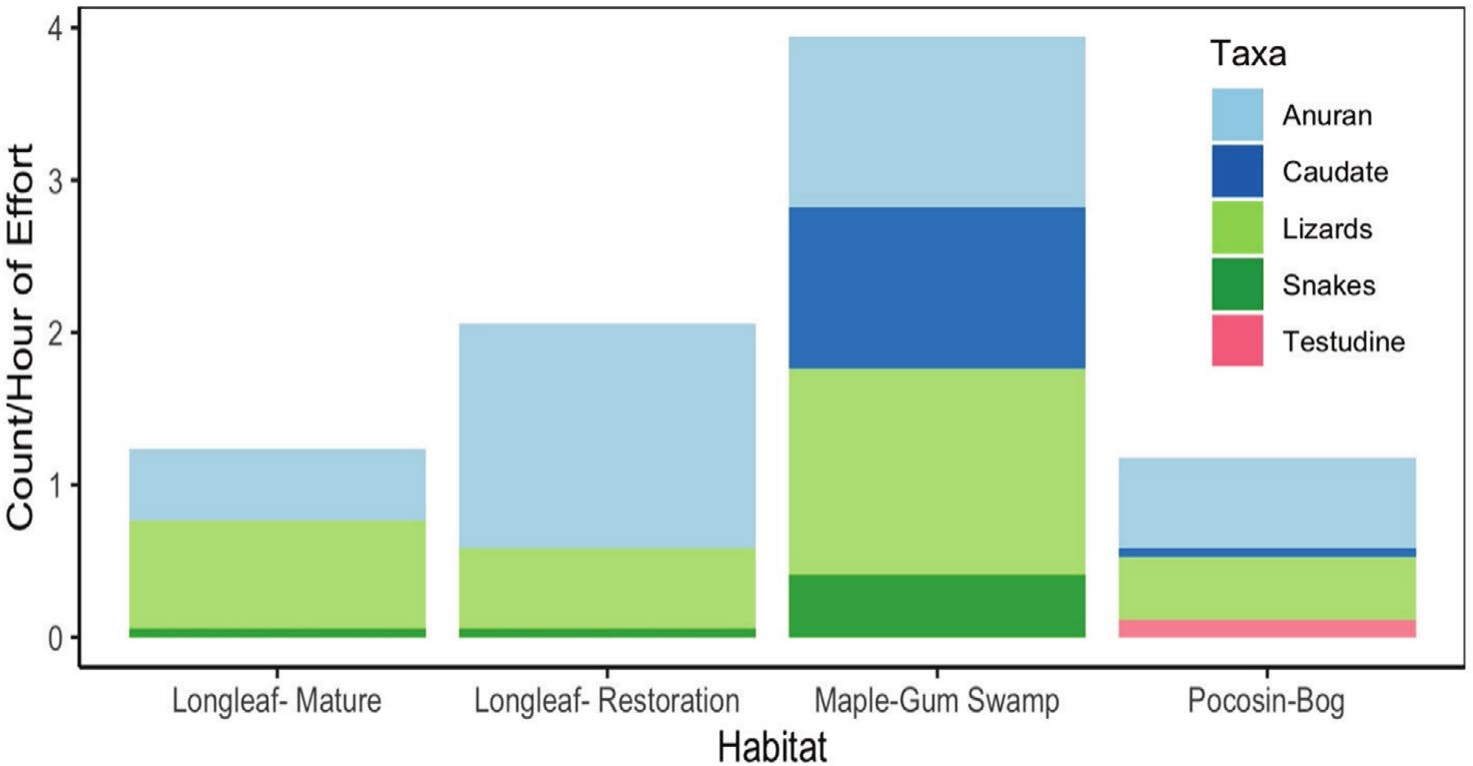
Comparison of count per hour of effort. Stacked bar graph is further divided by family of herpetofauna taxa.

**FIGURE 4:**
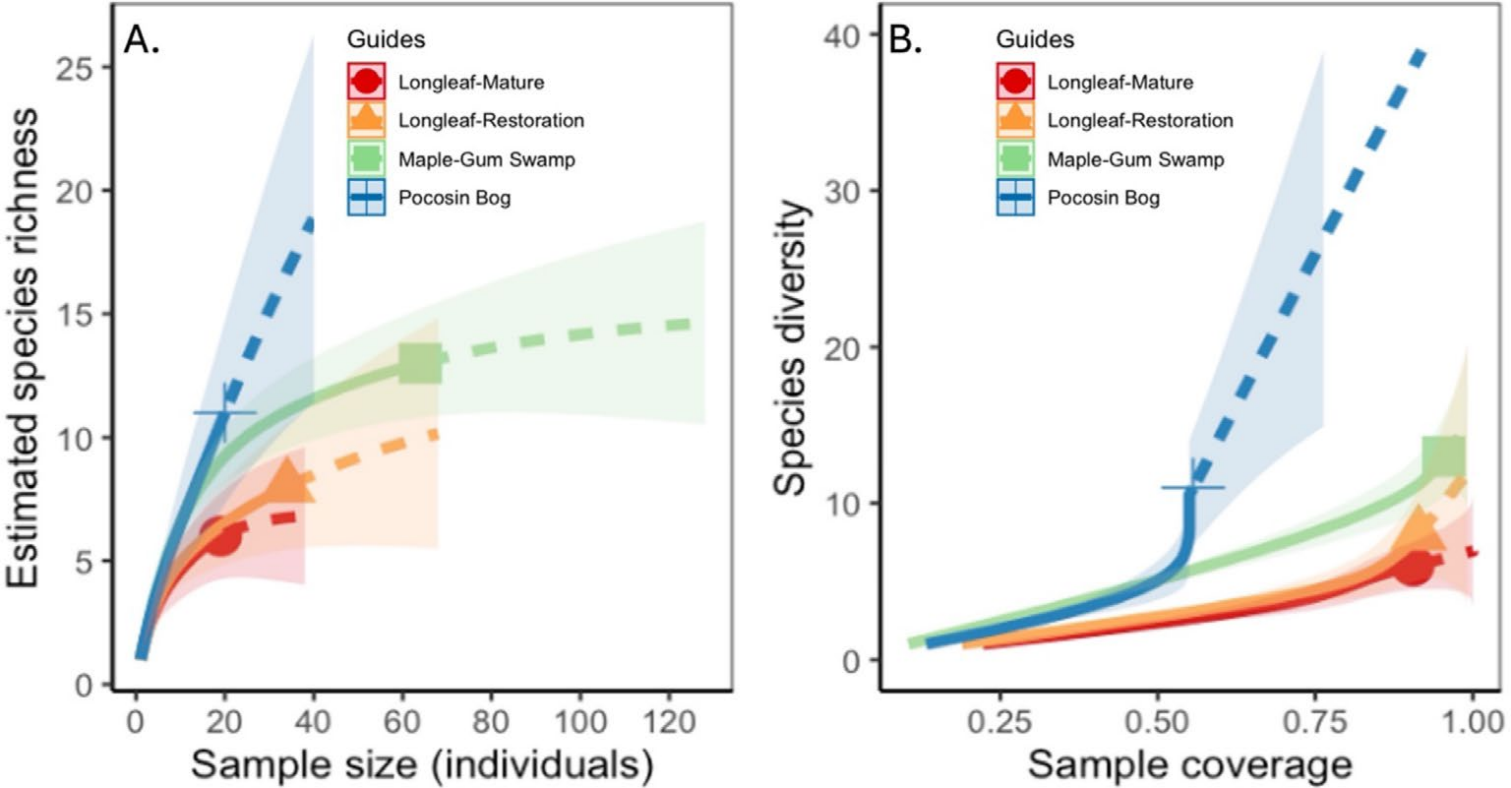
Estimated species richness with species rarefaction curve (A) and species diversity with species coverage curve (B) (solid = interpolated, dashed = extrapolated) for each habitat type sampled (Longleaf‐Mature = Red; Longleaf‐Restoration = Orange; Maple‐Gum Swamp = Green; Pocosin Bog = Blue). Shaded area indicates 95% confidence interval.

**FIGURE 5:**
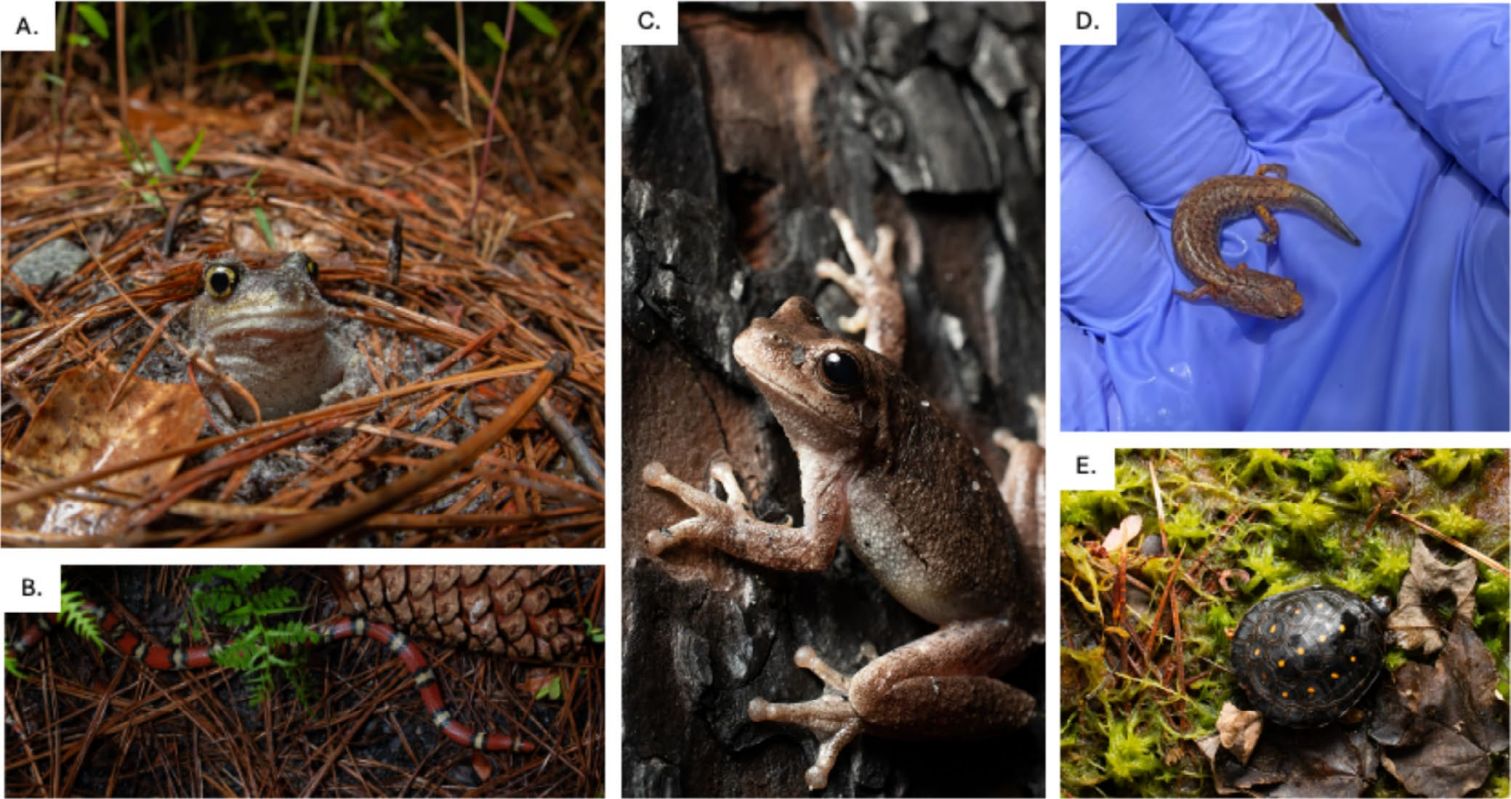
Photos of herpetofauna found within the study. (A) eastern spadefoot toad ( *Scaphiopus holbrookii* ), (B) northern scarlet snake ( *Cemophora coccinea copei* ), (C) pine woods tree frog ( *Dryophytes femoralis* ), (D) four‐toed salamander ( *Hemidactylium scutatum* ), (E) spotted turtle ( *Clemmys guttata* ). Images: J. Jones.

Our study found 25 herpetofauna species across the four focal habitats surveyed and 19 other species found incidentally on the two properties (Table 1; Table S2). The greatest number of species per effort was found in the maple‐gum swamp habitat, with the lowest number in the mature longleaf pine and pocosin‐bog (Figure 3). Estimated species richness was also highest in the maple‐gum swamp and lowest in the mature longleaf pine, but the pocosin‐bog habitat maintained the second estimated highest species richness (Figure 4A; Table 1). Anurans and lizards were the most common taxa found regardless of habitat, with the southern toad ( *Anaxyrus terrestris* ), pine woods tree frog ( *Dryophytes femoralis* ), and eastern fence lizard ( *Sceloporus undulatus* ) found in all habitats (Figure 3; Table 1). While most species found in our study are considered widespread generalists, several are further categorized as pinewoods or fire‐tolerant species (per Guyer and Bailey 1993) (Table 1, Figure 5), namely, the pinewoods tree frog ( *D. femoralis* ), the eastern spadefoot toad ( *Scaphiopus holbrookii* ), the eastern fence lizard ( *S. undulatus* ), the northern scarlet snake ( *Cemophora coccinea copei* ), and the six lined racerunner ( *Aspidoscelis sexlineatus* ). Additionally, the eastern slender glass lizard ( *Ophisaurus attenuatus longicaudus* ), also a pinewoods adapted species, was found outside of our four focal areas on the property (Table S1). Most of these pinewoods or fire‐tolerant species were found in both longleaf habitats and at least one adjacent habitat, with only the northern scarlet snake and the six lined racerunner found solely in the mature and restoration longleaf habitats, respectively. Sampling coverage highlights high levels of coverage for the maple‐gum swamp, mature longleaf, and restoration longleaf sites (> 0.85), but much lower levels of coverage in the pocosin‐bog (~0.60) (Figure 4B).

**TABLE 1:** List of all herpetofauna found in surveyed habitats with total number observed from February to July 2023.

Scientific name	Common name	Longleaf‐Mature	Longleaf‐Restoration	Maple‐Gum Swamp	Pocosin‐Bog
*Acris crepitans*	Eastern Cricket Frog			1	1
*Acris gryllus*	Southern Cricket Frog				1
*Anaxyrus terrestris*	Southern Toad	2	10	2	1
*Aspidoscelis sexlineatus sexlineatus* ^a^	Eastern Six‐Lined Racerunner		2		
*Carphophis amoenus amoenus*	Eastern Worm Snake			6	
*Cemophora coccinea copei* ^a^	Northern Scarlet Snake	1			
*Clemmys guttata*	Spotted Turtle				1
*Diadophis punctatus edwardsii*	Northern Ringneck Snake			1	
*Gastrophryne carolinensis*	Eastern Narrow Mouth Toad		1		
*Hemidactylium scutatum*	Four‐Toed Salamander				1
*Dryophytes cinerea*	Green Tree Frog			1	
*Dryophytes femoralis* ^a^	Pine Woods Tree Frog	6	4	4	5
*Lithobates catesbeianus*	American Bullfrog			5	
*Lithobates clamitans*	Green Frog			6	
*Lithobates sphenocephalus utricularius*	Coastal Plains Leopard Frog		1		1
*Plestiodon spp*	Skink Spp.	2		3	
*Plestiodon fasciatus*	Five‐Lined Skink	1			
*Plestiodon inexpectatus*	Southeastern Five‐Lined Skink				1
*Plestiodon laticeps*	Broadhead Skink	2		8	
*Plethodon cinereus*	Redback Salamander			5	
*Plethodon chlorobyronis*	Atlantic Coast Slimy Salamander			13	
*Scaphiopus holbrookii* ^a^	Eastern Spadefoot Toad		9		1
*Sceloporus undulatus* ^a^	Eastern Fence Lizard	7	7	10	6
*Scincella lateralis*	Little Brown Skink			2	
*Terrapene carolina carolina*	Eastern Box Turtle				1
*Thamnophis sirtalis sirtalis*	Eastern Garter Snake		1		

^a^ Pinewoods and/or fire‐tolerant species.

## 5 Discussion

We found 25 species of herpetofauna in the survey area and 19 incidental species in adjacent areas of the property, with most species being considered generalists but several considered either pinewoods or fire‐tolerant species. The greatest number of individuals per unit effort and highest estimated species richness was found in the maple‐gum swamp, and the fewest and least diverse group in the mature longleaf pine forest. Most species found, regardless of habitat, were anurans and lizards, while testudines were the least commonly found. Species diversity coverage was high for all sites except for the pocosin‐bog, where sampling continued to find additional species.

While many of the species found are considered generalists (e.g., southern Toad‐ *Anaxyrus terrestris* ), several pinewoods and fire‐tolerant species that prefer sandy soils and pinewoods (Guyer and Bailey 1993) were found in all habitats (e.g., pine woods tree frog— *D. femoralis* , eastern spadefoot toad— *A. terrestris* , eastern fence lizard— *S. undulatus* ). This is likely due to prescribed burns in all habitats or adjacent portions, the usage of multiple habitats, and the high prevalence of pine species (*Pinus* spp.‐ *See methods*) in all sampling sites. Further, much of the landscape maintains sandy soils to some degree with sandy Leon‐Chipley or loamy Alaga soils present (Soil Survey Staff 2024) and prescribed burns further maintaining mineral soils at or near the surface (DeFeo et al. 2025). Additionally, a lack of certain generalists in both the longleaf and wetland habitats, such as the commonly occurring green tree frog (*Droyphytes cinereus*) (Mitchell and Reay 1999), may open niches for pinewoods‐adapted species like the pinewood tree frog ( *D. femoralis* ) in all areas.

Species of note in the overall system include the spotted turtle ( *Clemmys guttata* ) found in the pocosin‐bog, an IUCN endangered species, and the eastern box turtle ( *Terrapene carolina carolina* ), a listed vulnerable species. Additionally, the northern scarlet snake and eastern spadefoot toad are listed as “Moderate Conservation Need” in Virginia (Virginia Department of Game and Inland Fisheries 2015). The eastern slender glass lizard ( *Ophisaurus attenuatus longicaudus* ) and little grass frog ( *Pseudacris ocularis* ) are also species of note, as they were both found in areas just outside the established mature longleaf unit and both listed as “Moderate Conservation Need”, as well as the oak toad ( *Anaxyrus quercicus* ) which is listed as a species of “Very High Conservation Need” (Virginia Department of Game and Inland Fisheries 2015). Finally, the four‐toed salamander (*Demidactylium scutatum*) and eastern cricket frog ( *Acris crepitans* ) are believed to be new county records for Isle of Wight County, Virginia (Virginia Herpetological Society 2025). Despite some species only being found incidentally in adjacent, non‐focal areas, it is critical to consider these species as they may use our study areas either intermittently or we did not detect them during our study; their proximal position may still indicate potential usage.

The maple‐gum swamp and the pocosin‐bog maintained the highest estimated species richness and species diversity of all sampled habitats, with frogs, salamanders, and turtles contributing many wetland‐specific observations. These findings mirror other studies that also found a similar trend of longleaf adjacent wetlands habitats maintaining greater diversity and abundance (Camper 2005). Flooding, at or above the surface, and moist soils were common throughout the entire sampling period from February to July in both the maple‐gum swamp and pocosin‐bog (pers. obs.). The maple‐gum swamp not only had highly moist conditions but was located within a broader slough that has intermittent connectivity to other nearby wetland features (e.g., road‐side ditches, ponds) (Markle et al. 2018). Further, the pocosin‐bog is one of several on the property (Frost and Musselman 1987) and may serve as refugia during fires and drought along with the maple‐gum swamp (Camper 2005). Our sampling coverage analysis highlights that our sampling protocol may still not have found all species present in the pocosin‐bog, especially compared to other habitat types. Habitat usage may also be linked to food resources, with both the maple‐gum swamp and pocosin‐bog maintaining markedly different vegetation complexity (Humphrey et al. 1999) compared to their surrounding environments, and in comparison to the far more open longleaf pine habitats sampled (Royal et al. 2022). Capture rates were highest in the maple‐gum swamp compared to all other systems, again similar to other studies (Camper 2005). As recaptures were not excluded from subsequent visits, capture counts across habitat types do not indicate individual abundance per se. In these cases, repeated captures throughout the season instead represent the consistent viability of a habitat type. Many species were found—regardless of their frequency—utilizing multiple or all the habitats studied, which may have important implications for managing habitat heterogeneity and maintaining a mosaic of habitat patches with restoration and conservation efforts (Law and Dickman 1998; Royal et al. 2022; Westaway et al. 2024).

When comparing mature and restored longleaf habitats, similar estimated species richness was seen, but overlap in species was limited. Potential explanations include greater understory cover and more diverse microhabitats, with a higher understory canopy in the restored longleaf habitat (pers. obs.) (Royal et al. 2022). The restored longleaf habitat also received a prescribed burn in April of the sampling year. Only the northern scarlet snake was found exclusively in the mature longleaf habitat, and only the six‐lined racerunner was exclusively found in the restored longleaf habitat, an indicator of fire‐adapted systems (Litt et al. 2001). Other studies have shown that more open canopies of mature longleaf stands and reduced midstory cover result in lower severity fires as well as greater opportunities for thermoregulation and certain food sources (Royal et al. 2022), but this may be specific for open habitat specialists (Litt et al. 2001).

Our work provides an initial examination of herpetofauna species in the Zuni Pine Barrens, at the latitudinal ecotone of longleaf pine, and highlights both species richness and diversity in this landscape mosaic. While only a limited number of pinewoods and fire‐tolerant species were found, our study provides a critical starting point for future scientific inquiry and an initial examination of herpetofauna in these systems that are currently undergoing substantial restoration efforts. This work showcases novel assemblages of fauna at the latitudinal limit of longleaf within a patchy ecotone and showcases how future restoration efforts should consider heterogeneity within the broader landscape to enhance species richness and community resilience. Further studies are needed to explore the complexities of future climate change, futuristic restoration, mosaic importance, and the impacts of fire regime and severity on herpetofauna at this and other longleaf pine latitudinal ecotones.

## Author Contributions

**Julianne Jones:** conceptualization (equal), data curation (equal), formal analysis (equal), funding acquisition (lead), investigation (lead), methodology (equal), project administration (lead), visualization (equal), writing – original draft (equal), writing – review and editing (equal). **Dylan Bryant:** investigation (equal), methodology (equal), writing – review and editing (equal). **Erik Yando:** conceptualization (equal), data curation (equal), formal analysis (equal), funding acquisition (supporting), investigation (supporting), methodology (equal), project administration (equal), resources (lead), supervision (lead), visualization (equal), writing – original draft (equal), writing – review and editing (equal).

## Conflicts of Interest

The authors declare no conflicts of interest.

## Supporting information

Table S1.

Table S2.

## Data Availability

All data not within the manuscript is publicly available on Github (Jones et al. 2025; 10.5281/zenodo.16634296, https://doi.org/10.5281/zenodo.15213926).
