# Supplementary material for: Herpetofauna Richness, Diversity, and Occurrence at the Northern Ecotone of Longleaf Pine
Source: Ecol Evol. 2025 Aug 31;15(9):e72041. doi: 10.1002/ece3.72041 (PMC12399407; doi:10.1002/ece3.72041)
Supplement: Supplementary file 2 — Table S2. [file ECE3-15-e72041-s001.docx]

**Table S2: Herpetofauna list of Blackwater Ecological Preserve and Antioch Pines Natural Area Preserve. Total of 44 species in all sampled habitats and in other adjacent areas within the preserve from February to July 2025. * County Record; ** Indicates species of concern, as listed on the Virginia Wildlife Action Plan**

**Anurans (Frogs & Toads)**

Eastern Narrow-mouthed Toad (*Gastrophryne carolinensis*)

Eastern Spadefoot** (*Scaphiopus holbrookii*)

Pinewoods Treefrog (*Dryophytes femoralis*)

Green Tree Frog (*Dryophytes cinerea*)

Cope’s Gray Treefrog (*Dryophytes chrysoscelis*)

Squirrel Tree Frog (*Dryophytes squirella*)

Spring Peeper (*Pseudacris crucifer*)

Brimley’s Chorus Frog (*Pseudacris brimleyi*)

Little Grass Frog** (*Pseudacris ocularis*)

Southern Toad (*Anaxyrus terrestris*)

American Bullfrog (*Lithobates catesbeaianus*)

Green Frog (*Lithobates clamitans*)

Coastal Plains Leopard Frog (*Lithobates sphenocephalus utricularius*)

Southern Cricket Frog (*Acris gryllus*)

Eastern Cricket Frog* (*Acris crepitans*)

Oak Toad** (*Anaxyrus quercicus*)

**Salamanders**

Two-Toed Amphiuma (*Amphiuma means*)

Redback salamander (*Plethodon cinereus*)

Atlantic Coast Slimy Salamander (*Plethodon chlorobryonis*)

Four-toed Salamander* (*Hemidactylium scutatum*)

Southern Two-lined Salamander (*Eurycea cirrigera*)

**Lizards**

Eastern Fence Lizard (*Sceloporus undulatus*)

Eastern Six-lined Race Runner (*Aspidoscelis sexlineatus sexlineatus*)

Southeastern Five-lined Skink (*Plestiodon inexpectatus*)

Common Five-Lined Skink (*Plestiodon fasciatus*)

Broadhead Skink (*Plestiodon laticeps*)

Little Brown Skink (*Scincella lateralis*)

Eastern Slender Glass Lizard (*Ophisaurus attenuatus longicaudus*)

**Snakes**

Northern Black Racer (*Coluber constrictor constrictor*)

Eastern Rat Snake (*Pantherophis quadrivittatus*)

Rough Green Snake (*Opheodrys aestivus aestivus*)

Northern Cottonmouth (*Agkistrodon piscivorus*)

Eastern Copperhead (*Agkistrodon contortrix*)

Northern Scarlet Snake** (*Cemophora coccinea copei*)

Plain-bellied Watersnake (*Nerodia erythogaster*)

Eastern Worm Snake (*Carphophis amoenus amoenus*)

Northern Ringneck (*Diadophis punctatus edwardsii*)

Southern Ringneck (*Diadophis punctatus punctatus*)

Dekays Brown Snake (*Storeria dekayi*)

Eastern Garter Snake (*Thamnophis sirtalis sirtalis*)

**Turtles**

Painted Turtle (*Chrysemys picta picta*)

Eastern Box Turtle** (*Terrapene carolina carolina*)

Spotted Turtle** (*Clemmys guttata*)

Common Snapping Turtle** (*Chelydra serpentina*)
